# Supplementary material for: Neighborhood cohesion and violence in Port-au-Prince, Haiti, and their relationship to stress, depression, and hypertension: Findings from the Haiti cardiovascular disease cohort study
Source: PLOS Glob Public Health. 2022 Jul 27;2(7):e0000503. doi: 10.1371/journal.pgph.0000503 (PMC9937441; doi:10.1371/journal.pgph.0000503)
Supplement: S1 Text — (DOCX) [file pgph.0000503.s001.docx]

**Supporting Information**

**Title**: Neighborhood cohesion and violence in Port-au-Prince, Haiti, and their relationship to cardiovascular risk factors of stress, depression, and blood pressure

**Authors**:

Lily D Yan ^1,2^, Margaret L McNairy^1,2^, Jessy G Dévieux^3^, Jean Lookens Pierre^4^, Eliezer Dade^4^, Rodney Sufra^4^, Linda M Gerber^5^, Nicholas Roberts^1,2^, Stephano St-Preux^4^, Rodolphe Malebranche^6,7^, Miranda Metz^1,2^, Olga Tymejczyk^8^, Denis Nash^8^, Marie Deschamps^4^, Monica M Safford^1^, Jean W Pape^2,4^, Vanessa Rouzier^2,4^

**Affiliations:**

1. Division of General Internal Medicine, Department of Medicine, Weill Cornell Medicine, New York, New York, USA

2. Center for Global Health, Department of Medicine, Weill Cornell Medicine, New York, New York, USA

3. Department of Health Promotion and Disease Prevention, Robert Stempel College of Public Health and Social Work, Florida International University, Miami, FL, USA.

4. Haitian Group for the Study of Kaposi's Sarcoma and Opportunistic Infections (GHESKIO), Port-au-Prince, Haiti

5. Department of Population Health Sciences, Weill Cornell Medicine, New York, NY, USA.

6. Collège Haïtien de Cardiologie, Port-au-Prince, Haiti

7. Medicine and Pharmacology, Université d'État d'Haïti, Port-au-Prince, Haiti

8. City University of New York Institute for Implementation Science in Population Health, New York, NY, USA

**Corresponding Author:**

Lily D Yan, MD MSc

Instructor of Medicine

Center for Global Health and Division of General Internal Medicine

Weill Cornell Medicine

402 East 67th Street, 2nd floor

NY, NY 10065

Telephone: (646) 962-8140, Fax: (646) 962-0285

[liy9032@med.cornell.edu](mailto:liy9032@med.cornell.edu)

**Figure A**: Flow diagram


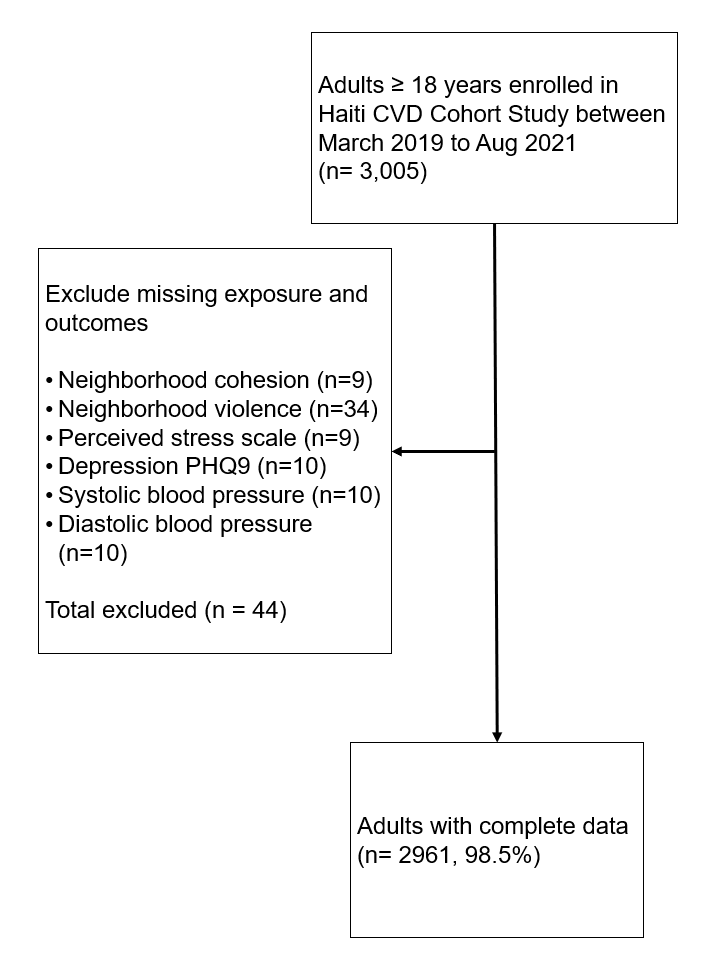


**Figure B**: Violence and enrollment


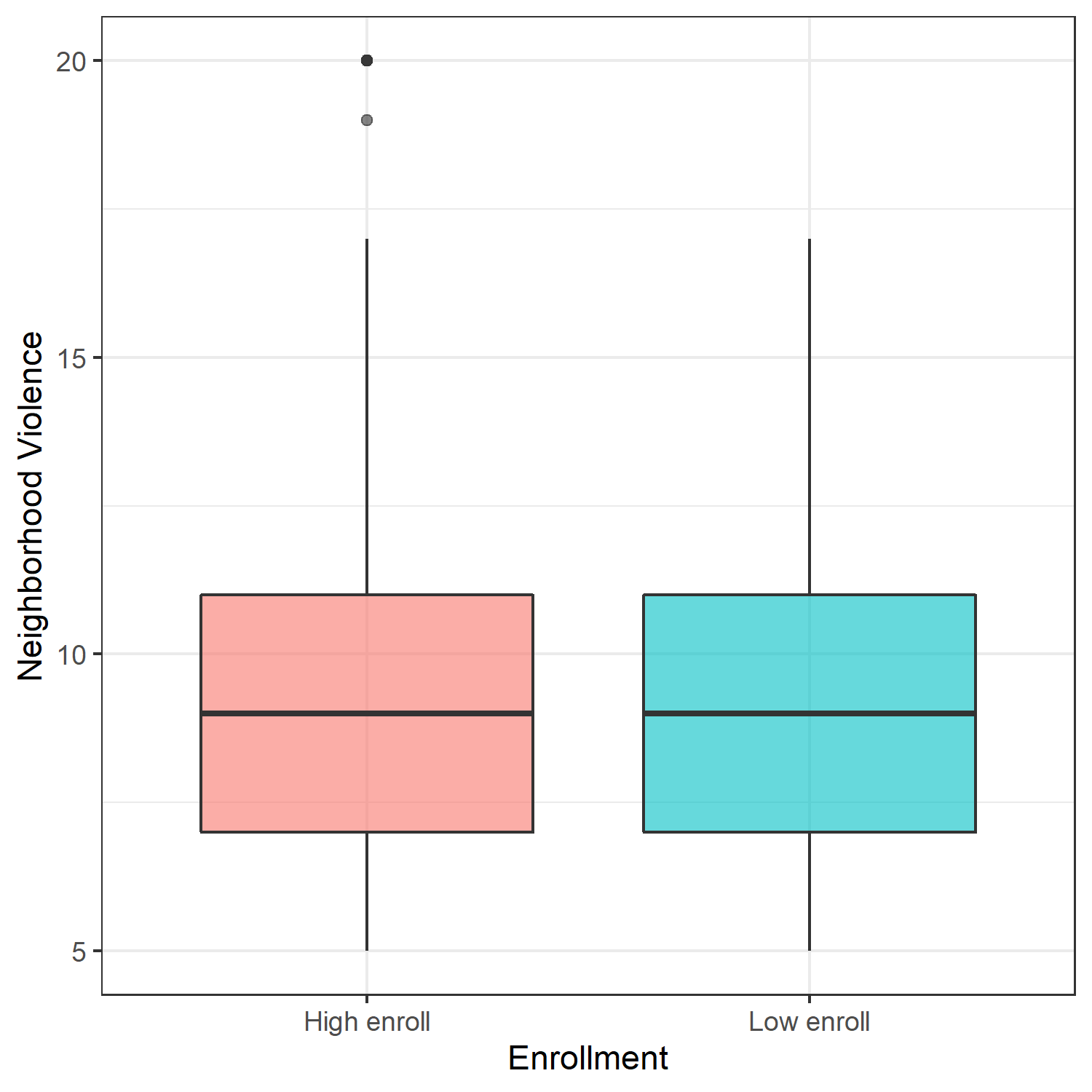


Legend: Boxplot of neighborhood violence score on Y axis, between high enrollment weeks (weeks with more than median number of enrollments per week) and low enrollment weeks (weeks with less than or equal to median number of enrollments per week). Median is horizontal line, interquartile range is box size. Whiskers represent 1.5 times the interquartile range, with more extreme values plotted as points.

**Table A: Adapted Questionnaires**

| **Neighborhood Collective Efficacy Scale**  Adapted from Browning 2002 | **Haitian Creole Translation** | **Answers** | **Haitian Creole Translation** |
| --- | --- | --- | --- |
| People in my neighborhood are willing to help their neighbors | Moun nan katye mwen vle ede vwazen yo | Strongly agree (5), Agree, Neutral, Disagree, Strongly disagree (1) | Dakò Dakò (5),  Byen Dakò,  Ni dakò ni pa dakò,  Pa dakò,  Pa dakò nèt (1) |
| This is a neighborhood where people are very united | Sa se yon katye kote moun yo ini anpil | Strongly agree (5), Agree, Neutral, Disagree, Strongly disagree (1) | Dakò Dakò (5),  Byen Dakò,  Ni dakò ni pa dakò,  Pa dakò,  Pa dakò nèt (1) |
| People in the neighborhood can be trusted | Mwen ka fè moun ki nan katye sa konfyans | Strongly agree (5), Agree, Neutral, Disagree, Strongly disagree (1) | Dakò Dakò (5),  Byen Dakò,  Ni dakò ni pa dakò,  Pa dakò,  Pa dakò nèt (1) |
| People in the neighborhood don’t get along | Moun ki nan katye sa pa antann yo youn ak lòt | Strongly agree (1), Agree, Neutral, Disagree, Strongly disagree (5) | Dakò Dakò (1),  Byen Dakò,  Ni dakò ni pa dakò,  Pa dakò,  Pa dakò nèt (5) |
| People in the neighborhood don’t share the same values | Moun ki nan katye sa pa pataje menm valè yo | Strongly agree (1), Agree, Neutral, Disagree, Strongly disagree (5) | Dakò Dakò (1),  Byen Dakò,  Ni dakò ni pa dakò,  Pa dakò,  Pa dakò nèt (5) |

| **City Stress Inventory**  Adapted from Ewart 2002 | **Haitian Creole Translation** | **Answers** | **Haitian Creole Translation** |
| --- | --- | --- | --- |
| In the past 12 months, I have heard adults arguing loudly or arguing in my neighborhood | Nan denye 12 mwa ki sot pase yo, mwen te tande granmoun ki te diskite fò oubyen te fè kont nan katye mwen an. | Never (1),  One time,  A few times,  Often (4) | Jamè (1),  Yon fwa,  Kèk fwa,  Anpil fwa,  Refize reponn (4) |
| In the past 12 months, someone in my family or a friend has been the victim of theft | Nan denye 12 mwa ki sot pase yo, yon moun nan fanmi w oswa yon zanmi w te viktim nan men volè. |  |  |
| In the past 12 months, me or a family member or a friend has been attacked or beaten | Nan denye 12 mwa ki sot pase yo, yo te atake oubyen bat oumenm oubyen yon moun nan fanmi ou oswa yon zanmi w. |  |  |
| In the past 12 months, me or a family member or a friend has been stabbed or shot | Nan denye 12 mwa ki sot pase yo, yo te ponyade oubyen tire oumenm oubyen yon moun nan fanmi ou oswa yon zanmi w. |  |  |
| In the past 12 months, I have heard shooting in my neighborhood | Nan denye 12 mwa ki sot pase yo, konbyen fwa ou te tande tire nan katye w? |  |  |

| **Perceived Stress Scale**  Adapted from Cohen 1983 | **Haitian Creole Translation** | **Answers** | **Haitian Creole Translation** |
| --- | --- | --- | --- |
| In the past month, I felt a lack of control | Nan dènye mwa ki sot pase a, konbyen fwa ou te santi ou pat kapab kontwole bagay enpòtan nan lavi ou? | Never (0),  Almost never,  Sometimes,  Fairly often,  Very often (4) | Jamè (0),  Prèske jamè,  Pafwa,  Byen souvan,  Trè souvan (4) |
| In the past month, I felt confident that I can manage my personal problems | Nan dènye mwa ki sot pase a, konbyen fwa ou te santi ou gen konfyans sou kapasite w pou jere pwoblèm pèsonèl ou yo? |  |  |
| In the past month, things went my way | Nan dènye mwa ki sot pase a, konbyen fwa ou te santi bagay yo te ale jan ou ta renmen an? |  |  |
| In the past month, I felt my difficulties were piled too high to overcome | Nan dènye mwa ki sot pase a, konbyen fwa ou te santi difikilte yo te anpile tèlman wo ou pa t kapab sipote yo? |  |  |

| **Patient Health Questionnaire 9 (PHQ 9)**  Adapted from Kroenke 2001 | **Haitian Creole Translation** | **Answers** | **Haitian Creole Translation** |
| --- | --- | --- | --- |
| In the past two weeks, I’ve felt little interest or pleasure in things I usually enjoy | Pa prèske enterese oswa pran plezi pou fè okenn aktivite? | Not at all (0),  Several days,  More than half the days,  Nearly every day (3) | Pa ditou (0), Plizyè jou,  Plis pase matyè jou yo,  Prèske chak jou (3) |
| In the past two weeks, I’ve felt down, depressed, or hopeless | Santi w kagou, deprime oswa pèdi espwa? |  |  |
| In the past two weeks, I’ve been sleeping too much or too little | Gen difikilte pou dòmi oswa rete dòmi, oswa ou dòmi twòp? |  |  |
| In the past two weeks, I’ve felt tired or little energy | Santi w fatige oswa pa gen anpil enèji? |  |  |
| In the past two weeks, I’ve had poor appetite or overeating | Pa gen apeti oswa ou manje twòp? |  |  |
| In the past two weeks, I’ve felt bad about myself | Ou santi w mal nan ou menm, oswa ou santi ou echwe oubyen ou se echèk pou oumenm oswa fanmi w? |  |  |
| In the past two weeks, I’ve had trouble concentrating | Ou gen pwoblèm pou konsantre sou kèk bagay, tankou li jounal oswa gade televizyon, jwe kat oubyen jwe domino? |  |  |
| In the past two weeks, I’ve felt very slow or fidgety | Ap deplase oswa pale tèlman dousman lòt moun remake sa? Oswa nan sans kontrè - ou enève oswa ajite w ap vire tounen anpil plis pase nòmal? |  |  |
| In the past two weeks, I’ve had thoughts of suicide or self harm | Ou santi ou ta pi byen si ou te mouri oswa fè tèt ou mal? |  |  |

**Table B:** Association between neighborhood cohesion and prehypertension, multivariable Poisson regression.

|  | Model 3: Prehypertension vs Normotension | |
| --- | --- | --- |
|  | Prevalence Ratio  [95% CI] | p |
| Neighborhood Cohesion Tertile 1 | ref |  |
| Neighborhood Cohesion Tertile 2 | 1.02 [0.98, 1.05] | 0.33 |
| Neighborhood Cohesion Tertile 3 | 0.96 [0.93, 1.00]* | 0.03 |
| **Age, years** |  |  |
| 18-29 | ref |  |
| 30-39 | 1.06 [1.02, 1.1]* | <0.001 |
| 40-49 | 1.15 [1.1, 1.2]* | <0.001 |
| 50-59 | 1.24 [1.18, 1.31]* | <0.001 |
| 60+ | 1.3 [1.22, 1.38]* | <0.001 |
| **Male vs Female** | 1.11 [1.08, 1.14]* | <0.001 |
| **Income (daily)** |  |  |
| ≤1 USD / day | 1 [0.97, 1.04] | 0.82 |
| >1 USD / day | ref |  |
| **Education** |  |  |
| Primary or lower | ref |  |
| Secondary or higher | 0.94 [0.9, 0.98]* | <0.001 |
| **BMI, kg/m^2^** |  |  |
| Underweight, Normal, Overweight (<29.9) | ref |  |
| Obese (≥30.0) | 1.08 [1.04, 1.13]* | <0.001 |
| **Smoking** |  |  |
| Never/Former | ref |  |
| Current | 0.96 [0.89, 1.03] | 0.27 |
| **Alcohol intake** |  |  |
| ≤1 drink a day | ref |  |
| >1 drink a day | 1.02 [0.95, 1.1] | 0.55 |
| **Physical activity** |  |  |
| Low | 1.01 [0.98, 1.03] | 0.72 |
| Moderate-high | ref |  |
| **Fruit Vegetable Daily Intake** |  |  |
| <5 servings | ref |  |
| ≥5 servings | 0.88 [0.77, 1.02] | 0.08 |

**Table C:** Association between neighborhood violence and prehypertension, multivariable Poisson regression.

|  | Model 3: Prehypertension vs Normotension | |
| --- | --- | --- |
|  | Prevalence Ratio  [95% CI] | p |
| Neighborhood Violence Tertile 1 | ref |  |
| Neighborhood Violence Tertile 2 | 0.99 [0.95, 1.03] | 0.55 |
| Neighborhood Violence Tertile 3 | 0.98 [0.94, 1.02] | 0.29 |
| **Age, years** |  |  |
| 18-29 | ref |  |
| 30-39 | 1.06 [1.02, 1.1]* | <0.001 |
| 40-49 | 1.15 [1.1, 1.2]* | <0.001 |
| 50-59 | 1.23 [1.17, 1.3]* | <0.001 |
| 60+ | 1.3 [1.22, 1.38]* | <0.001 |
| **Male vs Female** | 1.1 [1.07, 1.14]* | <0.001 |
| **Income (daily)** |  |  |
| ≤1 USD / day | 1 [0.97, 1.04] | 0.89 |
| >1 USD / day | ref |  |
| **Education** |  |  |
| Primary or lower | ref |  |
| Secondary or higher | 0.94 [0.9, 0.98]* | <0.001 |
| **BMI, kg/m^2^** |  |  |
| Underweight, Normal, Overweight (<29.9) | ref |  |
| Obese (≥30.0) | 1.08 [1.04, 1.13]* | <0.001 |
| **Smoking** |  |  |
| Never/Former | ref |  |
| Current | 0.96 [0.89, 1.04] | 0.34 |
| **Alcohol intake** |  |  |
| ≤1 drink a day | ref |  |
| >1 drink a day | 1.02 [0.95, 1.1] | 0.55 |
| **Physical activity** |  |  |
| Low | 1.01 [0.98, 1.04] | 0.63 |
| Moderate-high | ref |  |
| **Fruit Vegetable Daily Intake** |  |  |
| <5 servings | ref |  |
| ≥5 servings | 0.88 [0.77, 1.01] | 0.07 |

**Table D:** Association between neighborhood cohesion and mental or physical health outcomes, sex-stratified

|  | Model 1: Stress  Beta  [95% CI] | | Model 2: Moderate to Severe Depression  Prevalence Ratio  [95% CI] | | Model 3: HTN  Prevalence Ratio  [95% CI] | |
| --- | --- | --- | --- | --- | --- | --- |
|  | Female | Male | Female | Male | Female | Male |
| Neighborhood Cohesion Tertile 1 | ref | ref | ref | ref | ref | ref |
| Neighborhood Cohesion Tertile 2 | 0.12  [-0.16, 0.4] | 0.21  [-0.15, 0.58] | 0.96  [0.93, 1]* | 0.98  [0.95, 1.02] | 0.96  [0.84, 1.1] | 1.11  [0.92, 1.34] |
| Neighborhood Cohesion Tertile 3 | -0.32  [-0.69, 0.04] | -0.54  [-0.94, -0.14]* | 1  [0.96, 1.04] | 0.97  [0.94, 1.01] | 0.95  [0.81, 1.13] | 1.06  [0.86, 1.29] |
| **Age, years** |  |  |  |  |  |  |
| 18-29 | ref | ref | ref | ref | ref | ref |
| 30-39 | 0.48  [0.07, 0.89]* | -0.35  [-0.83, 0.12] | 0.97  [0.93, 1.02] | 1.01  [0.97, 1.06] | 6.3  [3.12, 12.7]* | 5.47  [2.69, 11.1]* |
| 40-49 | 0.65  [0.22, 1.07]* | 0.13  [-0.33, 0.6] | 1  [0.95, 1.05] | 1.03  [0.99, 1.08] | 14.5  [7.38, 28.6]* | 12.5  [6.49, 23.9]* |
| 50-59 | 0.22  [-0.23, 0.67] | 0  [-0.55, 0.54] | 0.97  [0.92, 1.03] | 1.01  [0.97, 1.06] | 22.5  [11.4, 44.4]* | 17.7  [9.28, 33.9]* |
| 60+ | 0.69  [0.23, 1.14]* | 0.15  [-0.38, 0.69] | 0.99  [0.93, 1.06] | 1.04  [0.99, 1.1] | 26.4  [13.4, 52.2]* | 24.6  [12.9, 46.9]* |
| **Income (daily)** |  |  |  |  |  |  |
| ≤1 USD / day | 0.31  [0.05, 0.57]* | 0.14  [-0.22, 0.49] | 1.08  [1.05, 1.12]* | 1.08  [1.05, 1.11]* | 0.98  [0.86, 1.12] | 1.21  [0.99, 1.48] |
| >1 USD / day | ref | ref | ref | ref | ref | ref |
| **Education** |  |  |  |  |  |  |
| Primary or lower | ref | ref | ref | ref | ref | ref |
| Secondary or higher | -0.32  [-0.62, -0.01]* | -0.93  [-1.36, -0.5]* | 0.97  [0.93, 1.01] | 0.98  [0.95, 1.02] | 0.79  [0.67, 0.93]* | 0.86  [0.71, 1.04] |
| **BMI, kg/m^2^** |  |  |  |  |  |  |
| Underweight, Normal, Overweight (<29.9) | ref | ref | ref | ref | ref | ref |
| Obese (≥30.0) | -0.23  [-0.52, 0.05] | -0.41  [-1.17, 0.36] | 0.98  [0.94, 1.01] | 1.01  [0.94, 1.09] | 1.22  [1.07, 1.38]* | 1.45  [1.11, 1.91]* |
| **Smoking** |  |  |  |  |  |  |
| Never/Former | ref | ref | ref | ref | ref | ref |
| Current | 0.29  [-0.36, 0.93] | 0.54  [-0.15, 1.23] | 1.07  [0.97, 1.18] | 1.02  [0.95, 1.09] | 0.99  [0.71, 1.38] | 0.6  [0.33, 1.08] |
| **Alcohol intake** |  |  |  |  |  |  |
| ≤1 drink a day | ref | ref | ref | ref | ref | ref |
| >1 drink a day | 0.54  [-0.9, 1.99] | 0.37  [-0.25, 1] | 1.17  [1.03, 1.33]* | 1.06  [0.99, 1.14] | 2.08  [1.2, 3.6]* | 0.74  [0.47, 1.19] |
| **Physical activity** |  |  |  |  |  |  |
| Low | -0.51  [-0.76, -0.26]* | -0.04  [-0.35, 0.27] | 0.9  [0.87, 0.93]* | 0.98  [0.95, 1.01] | 1.03  [0.91, 1.17] | 1.17  [0.99, 1.38] |
| Moderate-high | ref | ref | ref | ref | ref | ref |
| **Fruit Vegetable Daily Intake** |  |  |  |  |  |  |
| <5 servings | ref | ref | ref | ref | ref | ref |
| ≥5 servings | 1  [0.09, 1.91]* | -1.22  [-2.75, 0.32] | 1.05  [0.87, 1.26] | 0.98  [0.95, 1.01] | 0.93  [0.41, 2.12] | 0  [0, 0] |

**Table E:** Association between neighborhood violence and mental or physical health outcomes, sex-stratified

|  | Model 1: Stress  Beta  [95% CI] | | Model 2: Moderate to Severe Depression  Prevalence Ratio  [95% CI] | | Model 3: HTN  Prevalence Ratio  [95% CI] | |
| --- | --- | --- | --- | --- | --- | --- |
|  | Female | Male | Female | Male | Female | Male |
| Neighborhood Violence Tertile 1 | ref | ref | ref | ref | ref | ref |
| Neighborhood Violence Tertile 2 | 0.11  [-0.18, 0.39] | -0.15  [-0.51, 0.21] | 1.04  [1.01, 1.08]* | 1.04  [1.01, 1.07]* | 0.86  [0.75, 1] | 1.01  [0.84, 1.22] |
| Neighborhood Violence Tertile 3 | 0.38  [0.02, 0.74]* | 0.14  [-0.3, 0.57] | 1.14  [1.1, 1.2]* | 1.08  [1.04, 1.12]* | 0.89  [0.75, 1.05] | 1.13  [0.91, 1.4] |
| **Age, years** |  |  |  |  |  |  |
| 18-29 | ref | ref | ref | ref | ref | ref |
| 30-39 | 0.46  [0.05, 0.87]* | -0.36  [-0.83, 0.12] | 0.97  [0.93, 1.02] | 1.01  [0.97, 1.05] | 6.3  [3.12, 12.7]* | 5.45  [2.68, 11.1]* |
| 40-49 | 0.66  [0.24, 1.08]* | 0.08  [-0.39, 0.55] | 1  [0.95, 1.05] | 1.03  [0.99, 1.08] | 14.5  [7.37, 28.7]* | 12.5  [6.52, 23.9]* |
| 50-59 | 0.22  [-0.24, 0.67] | -0.01  [-0.56, 0.54] | 0.97  [0.92, 1.03] | 1.02  [0.97, 1.07] | 22.4  [11.3, 44.1]* | 18.0  [9.4, 34.3]* |
| 60+ | 0.69  [0.23, 1.14]* | 0.17  [-0.37, 0.71] | 0.99  [0.93, 1.06] | 1.05  [0.99, 1.1] | 26.2  [13.2, 51.7]* | 24.9  [13.1, 47.4]* |
| **Income (daily)** |  |  |  |  |  |  |
| ≤1 USD / day | 0.25  [-0.02, 0.51] | 0.01  [-0.35, 0.37] | 1.07  [1.03, 1.1]* | 1.07  [1.04, 1.1]* | 1.01  [0.88, 1.16] | 1.18  [0.97, 1.44] |
| >1 USD / day | ref | ref | ref | ref | ref | ref |
| **Education** |  |  |  |  |  |  |
| Primary or lower | ref | ref | ref | ref | ref | ref |
| Secondary or higher | -0.34  [-0.64, -0.03]* | -0.91  [-1.35, -0.48]* | 0.97  [0.93, 1.01] | 0.98  [0.94, 1.02] | 0.8  [0.68, 0.94]* | 0.86  [0.71, 1.03] |
| **BMI, kg/m^2^** |  |  |  |  |  |  |
| Underweight, Normal, Overweight (<29.9) | ref | ref | ref | ref | ref | ref |
| Obese (≥30.0) | -0.24  [-0.52, 0.05] | -0.38  [-1.17, 0.4] | 0.98  [0.94, 1.01] | 1  [0.93, 1.08] | 1.21  [1.06, 1.37]* | 1.46  [1.11, 1.91]* |
| **Smoking** |  |  |  |  |  |  |
| Never/Former | ref | ref | ref | ref | ref | ref |
| Current | 0.3  [-0.35, 0.94] | 0.61  [-0.12, 1.33] | 1.07  [0.97, 1.18] | 1.01  [0.94, 1.09] | 1.02  [0.73, 1.41] | 0.6  [0.33, 1.09] |
| **Alcohol intake** |  |  |  |  |  |  |
| ≤1 drink a day | ref | ref | ref | ref | ref | ref |
| >1 drink a day | 0.44  [-0.98, 1.85] | 0.3  [-0.33, 0.93] | 1.13  [1, 1.28]* | 1.05  [0.98, 1.13] | 2.09  [1.19, 3.64]* | 0.74  [0.46, 1.18] |
| **Physical activity** |  |  |  |  |  |  |
| Low | -0.42  [-0.67, -0.17]* | -0.01  [-0.33, 0.3] | 0.92  [0.89, 0.95]* | 0.98  [0.95, 1.01] | 1.01  [0.89, 1.14] | 1.18  [1, 1.39] |
| Moderate-high | ref | ref | ref | ref | ref | ref |
| **Fruit Vegetable Daily Intake** |  |  |  |  |  |  |
| <5 servings | ref | ref | ref | ref | ref | ref |
| ≥5 servings | 1.05  [0.22, 1.89]* | -0.95  [-2.48, 0.57] | 1.08  [0.9, 1.31] | 0.98  [0.96, 1.01] | 0.92  [0.41, 2.06] | 0  [0, 0] |

# STROBE Statement—Checklist of items that should be included in reports of *cross-sectional studies*

|  | Item No | Recommendation | Page No |
| --- | --- | --- | --- |
| **Title and abstract** | 1 | (*a*) Indicate the study’s design with a commonly used term in the title or the abstract | 1 |
|  |  | (*b*) Provide in the abstract an informative and balanced summary of what was done and what was found | 2 |
| Introduction | | | |
| Background/rationale | 2 | Explain the scientific background and rationale for the investigation being reported | 3 |
| Objectives | 3 | State specific objectives, including any prespecified hypotheses | 3-4 |
| Methods | | | |
| Study design | 4 | Present key elements of study design early in the paper | 4 |
| Setting | 5 | Describe the setting, locations, and relevant dates, including periods of recruitment, exposure, follow-up, and data collection | 4 |
| Participants | 6 | (*a*) Give the eligibility criteria, and the sources and methods of selection of participants | 4 |
| Variables | 7 | Clearly define all outcomes, exposures, predictors, potential confounders, and effect modifiers. Give diagnostic criteria, if applicable | 5-6 |
| Data sources/ measurement | 8* | For each variable of interest, give sources of data and details of methods of assessment (measurement). Describe comparability of assessment methods if there is more than one group | 5-6 |
| Bias | 9 | Describe any efforts to address potential sources of bias | 5-7 |
| Study size | 10 | Explain how the study size was arrived at | 4 |
| Quantitative variables | 11 | Explain how quantitative variables were handled in the analyses. If applicable, describe which groupings were chosen and why | 5-6 |
| Statistical methods | 12 | (*a*) Describe all statistical methods, including those used to control for confounding | 7 |
|  |  | (*b*) Describe any methods used to examine subgroups and interactions | 7 |
|  |  | (*c*) Explain how missing data were addressed | 7 |
|  |  | (*d*) If applicable, describe analytical methods taking account of sampling strategy | 7 |
|  |  | (*e*) Describe any sensitivity analyses | 7 |
| Results | | | |
| Participants | 13* | (a) Report numbers of individuals at each stage of study—eg numbers potentially eligible, examined for eligibility, confirmed eligible, included in the study, completing follow-up, and analysed | 8 |
|  |  | (b) Give reasons for non-participation at each stage | 8 |
|  |  | (c) Consider use of a flow diagram | Fig S1 |
| Descriptive data | 14* | (a) Give characteristics of study participants (eg demographic, clinical, social) and information on exposures and potential confounders | 8 |
|  |  | (b) Indicate number of participants with missing data for each variable of interest | 8-9 |
| Outcome data | 15* | Report numbers of outcome events or summary measures | 8-9 |
| Main results | 16 | (*a*) Give unadjusted estimates and, if applicable, confounder-adjusted estimates and their precision (eg, 95% confidence interval). Make clear which confounders were adjusted for and why they were included | 8-9 |
|  |  | (*b*) Report category boundaries when continuous variables were categorized | 8-9 |
|  |  | (*c*) If relevant, consider translating estimates of relative risk into absolute risk for a meaningful time period | N/A |
| Other analyses | 17 | Report other analyses done—eg analyses of subgroups and interactions, and sensitivity analyses | 8--9 |
| Discussion | | | |
| Key results | 18 | Summarise key results with reference to study objectives | 9 |
| Limitations | 19 | Discuss limitations of the study, taking into account sources of potential bias or imprecision. Discuss both direction and magnitude of any potential bias | 12 |
| Interpretation | 20 | Give a cautious overall interpretation of results considering objectives, limitations, multiplicity of analyses, results from similar studies, and other relevant evidence | 9-12 |
| Generalisability | 21 | Discuss the generalisability (external validity) of the study results | 9-12 |
| Other information | | | |
| Funding | 22 | Give the source of funding and the role of the funders for the present study and, if applicable, for the original study on which the present article is based | submission system |
