# Supplementary material for: Neighborhood cohesion and violence in Port-au-Prince, Haiti, and their relationship to stress, depression, and hypertension: Findings from the Haiti cardiovascular disease cohort study
Source: PLOS Glob Public Health. 2022 Jul 27;2(7):e0000503. doi: 10.1371/journal.pgph.0000503 (PMC9937441; doi:10.1371/journal.pgph.0000503)
Supplement: Attachment — Submitted filename: Response to Reviewers v3.docx [file pgph.0000503.s002.docx]

Dr Maurizio Trevisan

Academic Editor

PLOS Global Public Health

MS ID: PGPH-D-22-00428
MS TITLE: Neighborhood cohesion and violence in Port-au-Prince, Haiti, and their relationship to stress, depression, and hypertension: findings from the Haiti Cardiovascular Disease Cohort Study

April 2022

Dear Dr Trevisan and Reviewers

We would like to thank you and the two reviewers for carefully reviewing our manuscript. We have revised the paper in response to the reviewers’ feedback, which we believe has strengthened the manuscript. We have included our responses to each reviewer and a revised manuscript.

Sincerely,

Lily Yan MD, MSc

Instructor in Medicine

Center for Global Health and Division of General Internal Medicine

**Weill Cornell Medicine**

402 East 67^th^ Street, 2^nd^ floor

NY, NY 10065

[liy9032@med.cornell.edu](mailto:liy9032@med.cornell.edu)

Reviewer Comments to the Author

Reviewer #1: Comments

- I am not sure depression variable is appropriate for the linear regression analysis. Figure 2d is showing skewed distribution. I am suggesting authors to consider the logistic regression analyses using known cutoff points for all the outcome variables (stress, depression, hypertension).
  - **RESPONSE**: Thank you for your comment. For hypertension, this is already analyzed as a categorical outcome using the WHO defined cutoff points of SBP >= 140, DBP >= 90, or patient self-report of taking medications. We have used Poisson regression with robust standard errors, instead of logistic regression, because the outcome of hypertension was common. In this scenario, Poisson regressions are preferred as they produce better unbiased estimates of risk ratios.
    - **FOR REFERENCE:** <https://bmcmedresmethodol.biomedcentral.com/articles/10.1186/s12874-018-0519-5>
  - For depression, we have made the change suggested, but with only 2 categories instead of 5. Using the standard cut-off points would result in 5 categories as shown in Figure 2d (no depression, mild depression, moderate, moderate-severe, severe). Given the multiple categories, we would have to use ordinal logistic regression. Due to the difficulty of interpretation with 5 categories of depression using these standard cut-offs, we have simplified the outcome of depression into 2 categories: none or mild vs moderate to severe depression. We have performed Poisson regression models to analyze depression as this categorical outcome.
    - **CHANGES:** *Table 3, Table 4 multiple changes for Depression, now as categorical outcome of Moderate to Severe vs Mild to None.*
    - Line 201: *Stress was analyzed as a continuous outcome, while depression was analyzed as a categorical outcome (moderate to severe depression vs none or mild). Hypertension was also analyzed as a categorical outcome (hypertension vs none).*
    - *Line 471: In unadjusted Poisson regression, perceived cohesion was associated with lower prevalence ratio of moderate-to-severe depression with participants in Tertile 2 versus Tertile 1 having an increased PR of 0.96 (95% CI 0.94 to 0.99). After adjustment it remained associated with a lower prevalence ratio of depression (T2 vs T1 PR 0.97, 95% CI 0.94 to 0.99).*
    - *Line 688: Perceived violence was associated with a higher prevalence ratio of moderate-to-severe depression without adjustment (T3 vs T1 PR 1.14, 95% CI 1.11 to 1.18). After adjustment, perceived violence was associated with higher prevalence of depression (T3 vs T1 PR 1.12, 95% CI 1.09 to 1.16).*
  - For stress, this scale is usually analyzed as a linear variable in the literature. There are no standard cutoff points for this scale. Due to this, we have elected to keep stress as a linear outcome, in line with prior research.
    - **FOR REFERENCE**
      - Warttig SL, Forshaw MJ, South J, White AK. New, normative, English-sample data for the Short Form Perceived Stress Scale (PSS-4). J Health Psychol. 2013;18(12):1617-1628. doi:10.1177/1359105313508346
      - Cohen S, Kamarck T, Mermelstein R. A global measure of perceived stress. J Health Soc Behav. 1983;24(4):385-396.
      - Stubbs B, Veronese N, Vancampfort D, Prina AM, Lin P-Y, Tseng P-T, et al. Perceived stress and smoking across 41 countries: A global perspective across Europe, Africa, Asia and the Americas. Sci Rep. 2017;7: 7597. doi:10.1038/s41598-017-07579-w
- Page 5 line 90, Authors described that the cohort study participants are selected by a multistage random sampling. Does it mean that cohort members represent entire residents of Port-au-Prince? More explanation is regarding sampling methods is needed instead of just citing another paper (ref 18). In addition, is there any kind of sampling weights for each study participants?
  - **RESPONSE**: Thank you for your comment. We have added details to this section. While the original design was meant to be population-representative of Port-au-Prince, due to shifting violence across the city during the study period, certain census blocks had to be excluded as it was not safe for study staff to conduct home visits there. For this reason, we were not able to generate sampling weights for each study participant. Thus, we use the term “population-based” instead of “population-representative”.
  - **CHANGES:** *Line 91: The sampling frame was created from census blocks as enumerated by the Institut Haitien de Statistique et d’Informatique, with exclusion of blocks experiencing political violence. Using Geographic Information Software, waypoints were randomly assigned across census blocks, with number of waypoints per block proportional to its estimated population. Study staff then used standardized procedures to select the closest residential building to each waypoint within a 50-m radius to approach for screening.*
- Neighborhood cohesion and violence seems to be negatively correlated and can be evaluated concurrently instead of independently. How about making a new variable based on the cohesion and violence information and evaluate whether there is any interaction between two variables. For example, study participants could be divided into participants with low cohesion + low violence (ref); participants with low cohesion + high violence; participants with high cohesion + low violence; participants with high cohesion + high violence.
  - **RESPONSE**: Thank you for this comment. We think that while there may be a negative correlation between neighborhood cohesion and neighborhood violence, there are distinct and separate mechanisms between each of these and the outcomes of stress, depression, and hypertension. Thus, it may not make sense from a theoretical perspective to combine the two as an interaction term. However, for thoroughness we have done what the Reviewer has requested.
  - As there are not standard cut-offs for either neighborhood cohesion and neighborhood violence (for “low” or “high”), we have generated tertiles based on the distribution of our data. We have generated the interaction term as requested by the Reviewer to create 9 categories (Cohesion T1 Violence T1, Cohesion T1 Violence T2, Cohesion T1 Violence T3….etc). The full multivariable models are shown below and show no consistent association between the new interaction term and the outcomes of stress, moderate-severe depression, or hypertension.
  - We think this method of handling neighborhood cohesion and violence is less intuitive, and actually obscures important trends seen in the original analysis. It is not possible in this presentation to see how the isolated effect of violence impacts the outcomes, for example. Thus, we have elected to keep the original analysis.

| Stress | estimate | conf.low | conf.high | p.value |
| --- | --- | --- | --- | --- |
| (Intercept) | 8.32 | 7.87 | 8.78 | 0 |
| interactionC1V1 | ref |  |  |  |
| interactionC1V2 | 0.01 | -0.4 | 0.42 | 0.96 |
| interactionC1V3 | 0.13 | -0.33 | 0.59 | 0.57 |
| interactionC2V1 | 0.1 | -0.28 | 0.48 | 0.62 |
| interactionC2V2 | 0.05 | -0.33 | 0.43 | 0.78 |
| interactionC2V3 | 0.59 | 0.13 | 1.06 | 0.01 |
| interactionC3V1 | -0.54 | -1.06 | -0.02 | 0.04 |
| interactionC3V2 | -0.41 | -0.84 | 0.01 | 0.05 |
| interactionC3V3 | -0.16 | -0.67 | 0.35 | 0.54 |
| agecat30-39 | 0.12 | -0.19 | 0.42 | 0.46 |
| agecat40-49 | 0.41 | 0.1 | 0.72 | 0.01 |
| agecat50-59 | 0.1 | -0.25 | 0.44 | 0.58 |
| agecat60+ | 0.47 | 0.12 | 0.81 | 0.01 |
| ind_sexmale | -0.8 | -1.01 | -0.58 | 0 |
| obeseObese | -0.25 | -0.51 | 0.02 | 0.07 |
| smokecurrentCurrent | 0.38 | -0.1 | 0.86 | 0.12 |
| alcoholMore than 1 drink a day (moderate or higher) | 0.28 | -0.31 | 0.87 | 0.34 |
| activityless or equal 150 min/week (low) | -0.29 | -0.49 | -0.09 | 0 |
| fruitveg5servMore than 5 servings fruit/veg per day | 0.63 | -0.25 | 1.51 | 0.16 |
| incomeless or equal 1 USD | 0.21 | -0.01 | 0.42 | 0.06 |
| eduSecondary or higher | -0.55 | -0.8 | -0.3 | 0 |

| ModSevereDepress | estimate | conf.low | conf.high | p.value |
| --- | --- | --- | --- | --- |
| (Intercept) | 1.18 | 1.12 | 1.25 | 0 |
| interactionC1V1 | ref |  |  |  |
| interactionC1V2 | 1.03 | 0.98 | 1.07 | 0.22 |
| interactionC1V3 | 1.13 | 1.07 | 1.19 | 0 |
| interactionC2V1 | 0.95 | 0.92 | 0.99 | 0.02 |
| interactionC2V2 | 1.03 | 0.98 | 1.07 | 0.24 |
| interactionC2V3 | 1.07 | 1.01 | 1.13 | 0.01 |
| interactionC3V1 | 0.99 | 0.94 | 1.05 | 0.82 |
| interactionC3V2 | 1.02 | 0.97 | 1.07 | 0.46 |
| interactionC3V3 | 1.1 | 1.04 | 1.16 | 0 |
| agecat30-39 | 0.99 | 0.96 | 1.02 | 0.53 |
| agecat40-49 | 1.02 | 0.98 | 1.05 | 0.38 |
| agecat50-59 | 0.99 | 0.96 | 1.03 | 0.76 |
| agecat60+ | 1.02 | 0.98 | 1.07 | 0.28 |
| ind_sexmale | 0.88 | 0.86 | 0.91 | 0 |
| obeseObese | 0.98 | 0.95 | 1.01 | 0.18 |
| smokecurrentCurrent | 1.04 | 0.98 | 1.1 | 0.24 |
| alcoholMore than 1 drink a day (moderate or higher) | 1.08 | 1.01 | 1.15 | 0.02 |
| activityless or equal 150 min/week (low) | 0.94 | 0.92 | 0.96 | 0 |
| fruitveg5servMore than 5 servings fruit/veg per day | 1.06 | 0.92 | 1.23 | 0.42 |
| incomeless or equal 1 USD | 1.07 | 1.04 | 1.09 | 0 |
| eduSecondary or higher | 0.97 | 0.94 | 1 | 0.08 |

| HTN | estimate | conf.low | conf.high | p.value |
| --- | --- | --- | --- | --- |
| (Intercept) | 0.03 | 0.02 | 0.04 | 0 |
| interactionC1V1 | ref |  |  |  |
| interactionC1V2 | 0.85 | 0.71 | 1.02 | 0.08 |
| interactionC1V3 | 0.93 | 0.75 | 1.15 | 0.51 |
| interactionC2V1 | 0.95 | 0.8 | 1.13 | 0.56 |
| interactionC2V2 | 0.92 | 0.77 | 1.09 | 0.32 |
| interactionC2V3 | 0.93 | 0.75 | 1.14 | 0.47 |
| interactionC3V1 | 0.89 | 0.71 | 1.12 | 0.33 |
| interactionC3V2 | 0.9 | 0.74 | 1.09 | 0.28 |
| interactionC3V3 | 0.97 | 0.78 | 1.21 | 0.81 |
| agecat30-39 | 5.79 | 3.53 | 9.49 | 0 |
| agecat40-49 | 13.2 | 8.26 | 21.11 | 0 |
| agecat50-59 | 19.65 | 12.31 | 31.38 | 0 |
| agecat60+ | 24.67 | 15.44 | 39.42 | 0 |
| ind_sexmale | 1.03 | 0.92 | 1.14 | 0.62 |
| obeseObese | 1.25 | 1.11 | 1.41 | 0 |
| smokecurrentCurrent | 0.82 | 0.61 | 1.1 | 0.19 |
| alcoholMore than 1 drink a day (moderate or higher) | 0.94 | 0.64 | 1.39 | 0.77 |
| activityless or equal 150 min/week (low) | 1.09 | 0.99 | 1.21 | 0.09 |
| fruitveg5servMore than 5 servings fruit/veg per day | 0.74 | 0.31 | 1.76 | 0.5 |
| incomeless or equal 1 USD | 1.07 | 0.96 | 1.2 | 0.24 |
| eduSecondary or higher | 0.81 | 0.72 | 0.92 | 0 |

- Detailed explanations regarding adjusted variables are needed. For example, how physical activities are categorized into low and moderate and high? What is low and high physical activities? How are servings defined? What is the difference between primary and secondary education is Haiti?
  - **RESPONSE**: Thank you for this suggestion. We have added a section under Methods, titled “Measurements”, to outline these adjusted variables.
  - **CHANGES:** *Line 118:* *Measurements*
  - *Sociodemographic data (age, sex, education, income) were collected using a standardized questionnaire at enrollment. We classified the level of education in two groups: primary school or lower versus secondary school or higher. Daily income was measured in Haitian Gourdes and converted into two categories of ≤1 USD versus >1 USD. Health behaviors (diet, smoking status, alcohol, physical activity) were collected using standardized WHO STEPs instruments 20.*
  - *Smoking status was ascertained from questions asking if the participant ever smoked tobacco, and if they currently smoke tobacco. Alcohol intake was grouped into ≤1 drink daily or >1 drink daily. Physical activity was determined from questions asking whether the participant did vigorous work or recreational activity for > 75 minutes a week, or moderate work or recreational activity for > 150 minutes a week. Participants were categorized as “moderate-high” if they reported yes to moderate or vigorous activity. We categorized diet by the median daily servings of fruit or vegetable intake, and categorizing it by < 5 servings a day versus ≥5 servings a day (the recommended limit by the WHO)20.*
  - *Clinical data including height, weight and BP were measured during a physical exam with a study nurse or physician at the time of study enrollment. Body mass index (BMI) was categorized using standard thresholds into underweight or normal (BMI ≤ 24.9 kg/m2), overweight (25.0-29.9 kg/m2) or obese (≥30.0 kg/m2).*
- Page 7 line 135: Detailed cutoff values should be presented.
  - **RESPONSE**: Thank you for your comment. We have added cutoff values for the tertiles for neighborhood cohesion and violence.
  - **CHANGES:** *Line 157: Based on the distribution of data, neighborhood cohesion and violence were categorized into tertiles (Cohesion: T1 5 to 14, T2 15 to 16, T3 17 to 25; Violence: T1 5 to 7, T2 8 to 10, T3 11 to 20).*
- Table 3 4 Considering very small amount of discussion regarding effects of adjusted variables on outcome variables, I am not sure coefficient for age, education, sex, BMI, and other confounder variables should be presented in the main Table.
  - **RESPONSE**: Thank you for this comment. We initially thought about moving the coefficients for the adjusted variables into the Supplemental online file. However, given the requested additional analysis from both reviewers (some of which will be included in the supplemental file), we have elected to keep the full table with all covariates as Table 3 and 4 rather than having only short tables in the main manuscript and full tables in the Supplement.

Reviewer #2:

Overall, this is an interesting study and well written manuscript, based on cross-sectional data from a population-based sample of adults in Port-au-Prince, Haiti. The authors examined cross-sectional associations of neighborhood cohesion and violence with a range of outcomes including perceived stress, depression, and hypertension prevalence.

Given the relatively unexplored setting, the present study may provide some novel evidence from an unstable, transitional setting, such as Haiti. They found higher violence associated with higher stress and depression, in line with previous evidence. There was no consistent association with hypertension. The relatively large sample size and random sampling are major strengths of the study. There are a few concerns, which are outline below. Specifically:

- Please clarify the participation and response rate of the random sample to rule out major selection bias in the study population.
  - **RESPONSE**: Thank you for this comment. We have added additional detail to the Methods about the multistage random sampling. In addition, to clarify this specific comment, out of 2,828 households initially recruited, 5,124 individuals were invited to GHESKIO for study screening, and 3,074 presented for study screening. 3,019 initiated informed consent and enrollment procedures, with 3,005 who completed enrollment. The remaining 14 participants signed informed consent but did not complete enrollment despite multiple attempts to re-contact.
  - **CHANGES:**
    - *Line 91: The sampling frame was created from census blocks as enumerated by the Institut Haitien de Statistique et d’Informatique, with exclusion of blocks experiencing political violence. Using Geographic Information Software, waypoints were randomly assigned across census blocks, with number of waypoints per block proportional to its estimated population. Study staff then used standardized procedures to select the closest residential building to each waypoint within a 50-m radius to approach for screening.*
- The percentage of missing data seems very low (only 1.5%), which is surprising. Can you please clarify the strategies implemented to mitigate this potential issue?
  - **RESPONSE**: Thank you for this comment. GHESKIO, the medical clinic where this research was conducted, has a 40 year history of conducting NIH sponsored clinical trials initially in HIV/TB, and now in cardiovascular disease research. During this time it has developed standardized and rigorous protocols to ensure high participant retention including a robust network of community health workers. Strategies implemented in this specific study include: phone calls every 3 months to all participants to ascertain vital status and symptom screening with referral to the clinic as needed, home visits every 6 months, weekly meetings with quality checks run on missing data and review of strategies to obtain missing data, involvement of the data team at GHESKIO in finding missing data, use of an electronic survey tool REDCap which facilitates real-time quality checks and reports on data issues.
  - In addition, prior to the initiation of the Haiti CVD Cohort, a pilot study was conducted during which many procedures related to recruitment, enrollment, retention, and missing data were developed and iterated upon.
- Hypertension was defined based on blood pressure values and self-report of medication. What about self-report of physician diagnosis?
  - **RESPONSE**: Thank you for this comment. Patient self-report of a physician diagnosis was not included in our definition of hypertension. We conducted extensive training and quality assurance around research-grade BP measurements in this study to ensure accurate measurement. We used automated-electronic cuffs (OMRON HEM 907), similar to those used in SPRINT. Participants rested for 5 minutes, then had 3 BPs measured, separated by 1 minute intervals, with the average of the last 2 BPs used for analysis.
  - In addition, our definition of hypertension is based on prior literature that uses cross-sectional data to define hypertension, please see the references below.
  - **FOR REFERENCE**
    - Geldsetzer, Pascal, Jennifer Manne-Goehler, Maja-Emilia Marcus, Cara Ebert, Zhaxybay Zhumadilov, Chea S Wesseh, Lindiwe Tsabedze, et al. “The State of Hypertension Care in 44 Low-Income and Middle-Income Countries: A Cross-Sectional Study of Nationally Representative Individual-Level Data from 1·1 Million Adults.” *The Lancet* 394, no. 10199 (August 24, 2019): 652–62. <https://doi.org/10.1016/S0140-6736(19)30955-9>.
    - Berry, Kaitlyn M., Whadi-Ah Parker, Zandile J. Mchiza, Ronel Sewpaul, Demetre Labadarios, Sydney Rosen, and Andrew Stokes. “Quantifying Unmet Need for Hypertension Care in South Africa through a Care Cascade: Evidence from the SANHANES, 2011-2012.” *BMJ Global Health* 2, no. 3 (2017): e000348. <https://doi.org/10.1136/bmjgh-2017-000348>.
- I would strongly encourage the authors to examine the same associations with prehypertension as an alternative outcome, which might provide additional novelty to the study.
  - **RESPONSE**: Thank you for this comment. We have added a sensitivity analyses using prehypertension vs normotension, instead of hypertension vs not hypertension, as the outcome. We have included these tables in the Supplement and added additional text to the manuscript.
  - **CHANGES:** *Line 204: As a sensitivity analysis, multivariable Poisson regression was used to examine the association between exposures and prehypertension as a categorical outcome, excluding people with hypertension.*
  - *Line 471: In a sensitivity analysis, perceived cohesion was associated with a lower prevalence ratio of prehypertension (T3 vs T1 PR 0.96, 95% CI 0.93 to 1.00) (S1 Text, Table B).*
  - *Line 676: Perceived violence was not associated with prehypertension (S1 Text Table C).*
  - *Supplemental online file, added Table B: Association between neighborhood cohesion and prehypertension, multivariable Poisson regression, and Table C: Association between neighborhood violence and prehypertension, multivariable Poisson regression.*
- Likewise, I would strongly encourage the authors to provide sex-stratified analyses, in line with current trends in cardiovascular epidemiology.
  - **RESPONSE**: Thank you for this comment. We have conducted sex-stratified analysis of Tables 3 and 4, included in the Supplemental Online data.
  - **CHANGES:** *Tables D and E in Supplemental Online data include sex-stratified multivariable analysis.*
  - *Line 252 In sex-stratified analyses, this relationship was true only among males (T3 vs T1 -0.54 score, 95% CI -0.94, -0.14) (S1 Text Table D).*
  - *Line 480: In sex-stratified analyses, the lower prevalence of depression was only seen in females (S1 Text Table D).*
  - *Line 537: In sex-stratified analyses, higher stress was only seen in females (T3 vs T1 + 0.38 score, 95% CI 0.02 to 0.74) (S1 Text Table E).*
  - *Line 742: The absence of this association was also seen in sex-stratified analyses (S1 Text Table E).*
  - *Line 825: In sex-stratified analyses, the strongest association between perceived violence and higher depression was seen in both females and males. The remaining associations were seen only in one biological sex. In males, perceived cohesion was associated with lower stress. In females, perceived cohesion was associated with lower depression and perceived violence with higher stress. This may suggest sex-specific mechanisms for how environmental factors affect mental health outcomes.*
- The authors often talk in terms of high or moderate neighborhood cohesion and violence. It is unclear the reference/range used to define these two parameters as high, moderate or low. Is this classification in relation to previous studies? Please clarify this issue.
  - **RESPONSE**: Thank you for this comment. These scores are not systematically categorized and usually kept as continuous variables, as violence/cohesion can be relative across contexts. We elected to use low, moderate, or high for neighborhood cohesion and violence as descriptive, qualitative terms based on equal-interval categories. In other words, for neighborhood cohesion (range 5 – 25), roughly equal-interval categories would be 5-12, 13-19, 20-25. For neighborhood violence (range 5-20), equal-interval categories would be 5-10, 11-15, 16-20. We have edited the first paragraph in the Discussion to try and clarify.
  - **CHANGES:** *Line 281: We found high levels of perceived neighborhood cohesion (median 15/25) coupled with moderate levels of perceived violence (9/20).*
- Among the study limitations, the authors should also acknowledge the cross-sectional design, as well as the lack of additional potential confounders, such as sleep patterns, which have been linked to both neighborhood characteristics and CVD risk.
  - **RESPONSE**: Thank you for this comment. We have added these suggestions to the study limitations section.
  - **CHANGES:** *Line 333: Limitations include the potential selection bias due to civil unrest, use of self-reported data, the cross-sectional study design, and potential omitted variable bias in the regressions such as lack of data on sleep patterns.*
